# Advancements in CHO metabolomics: techniques, current state and evolving methodologies

**DOI:** 10.3389/fbioe.2024.1347138

**Published:** 2024-03-27

**Authors:** Rita Singh, Eram Fatima, Lovnish Thakur, Sevaram Singh, Chandra Ratan, Niraj Kumar

**Affiliations:** ^1^ Translational Health Science and Technology Institute, Faridabad, India; ^2^ Jawaharlal Nehru University, New Delhi, India

**Keywords:** Chinese hamster ovary cells, metabolomics, bioprocess, cell engineering, growth, productivity, extraction, process design

## Abstract

**Background:** Investigating the metabolic behaviour of different cellular phenotypes, i.e., good/bad grower and/or producer, in production culture is important to identify the key metabolite(s)/pathway(s) that regulate cell growth and/or recombinant protein production to improve the overall yield. Currently, LC-MS, GC-MS and NMR are the most used and advanced technologies for investigating the metabolome. Although contributed significantly in the domain, each technique has its own biasness towards specific metabolites or class of metabolites due to various reasons including variability in the concept of working, sample preparation, metabolite-extraction methods, metabolite identification tools, and databases. As a result, the application of appropriate analytical technique(s) is very critical.

**Purpose and scope:** This review provides a state-of-the-art technological insights and overview of metabolic mechanisms involved in regulation of cell growth and/or recombinant protein production for improving yield from CHO cultures.

**Summary and conclusion:** In this review, the advancements in CHO metabolomics over the last 10 years are traced based on a bibliometric analysis of previous publications and discussed. With the technical advancement in the domain of LC-MS, GC-MS and NMR, metabolites of glycolytic and nucleotide biosynthesis pathway (glucose, fructose, pyruvate and phenylalanine, threonine, tryptophan, arginine, valine, asparagine, and serine, etc.) were observed to be upregulated in exponential-phase thereby potentially associated with cell growth regulation, whereas metabolites/intermediates of TCA, oxidative phosphorylation (aspartate, glutamate, succinate, malate, fumarate and citrate), intracellular NAD+/NADH ratio, and glutathione metabolic pathways were observed to be upregulated in stationary-phase and hence potentially associated with increased cell-specific productivity in CHO bioprocess. Moreover, each of technique has its own bias towards metabolite identification, indicating their complementarity, along with a number of critical gaps in the CHO metabolomics pipeline and hence first time discussed here to identify their potential remedies. This knowledge may help in future study designs to improve the metabolomic coverage facilitating identification of the metabolites/pathways which might get missed otherwise and explore the full potential of metabolomics for improving the CHO bioprocess performances.

## 1 Introduction

Chinese Hamster ovary (CHO) cells are the expression system of choice for production of over 70% of all recombinant biopharmaceutical proteins including monoclonal antibodies (like adalimumab, bezlotoxumab, brodalumab, etc.) and complex human proteins (*i.e.*, erythropoietin and clotting factors, etc.) due to their ability to grow fast to achieve high-density in protein-free and chemically defined culture media, perform human-like post-translational modifications, appropriate protein folding, high productivity and low susceptibility to human viral infections (Wurm, 2004; Lalonde and Durocher, 2017; Singh et al., 2023). However, the cost of such products is still high and requires significant improvements in the overall yield. To date, strategies like cell engineering, enriched culture media development and process optimization (biphasic culture approaches such as temperature shift and/or use of chemicals affecting cell cycle) (Trummer et al., 2006; Bollati-Fogolı, 2008; Kaisermayer et al., 2016; Ritacco et al., 2018; Huang et al., 2020; McHugh et al., 2020; Weng et al., 2020; Donaldson et al., 2022) have been employed to meet the increasing global demand at an affordable cost. As a result, CHO production cultures are currently able to achieve up to 10 g/L, which is 100-fold higher since 1980s. However, more improvement in the yield and quality are required to further reduce their cost and this can only be achieved by improving our understanding of CHO cell biology and their behaviour in bioprocess, thereby demanding greater efforts in this direction.

Multidimensional “omics” approaches like genomics, proteomics, transcriptomics and metabolomics have been proven to be powerful and complementary tools for exploratory research and improving the current knowledge (Jendoubi, 2021). To date, the potentials of genomics, transcriptomics and proteomics in CHO biology is being explored extensively (Jendoubi, 2021; Menyhárt and Győrffy, 2021). However, limited efforts have been made to understand the CHO cell metabolism in bioprocess (Lewis et al., 2016) and hence being targeted in this review.

**FIGURE 1:**
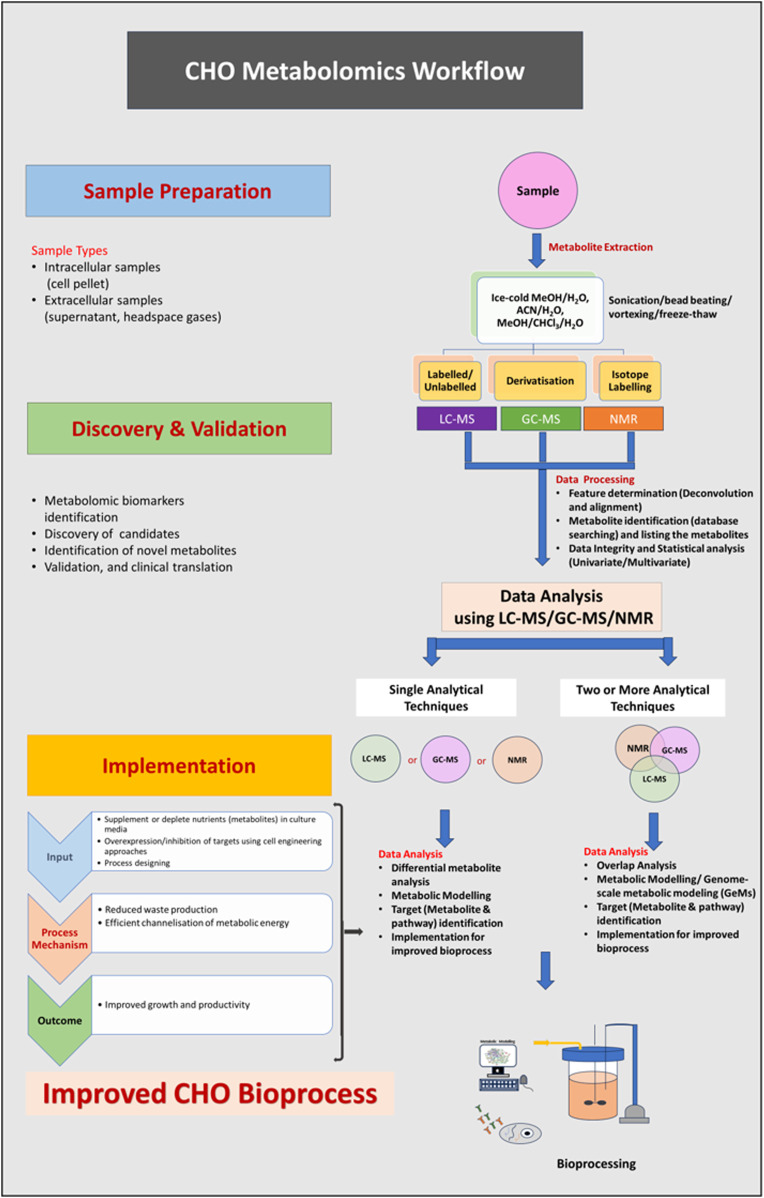
(Abstract Image)- A metabolomics workflow strategy to measure the differences in intracellular/extracellular metabolites of CHO cells. Sample preparation: culture cells in appropriate medium, collect the spent media and centrifuge to remove the cells/debris from spent media (if analyzing the metabolites in media) or collect cells (wash them twice), quench and extract the metabolites using analytical technique-compatible method. Discovery and validation: lyophilize the metabolites, reconstitute them before the run, and analyse using analytical techniques (LC-MS/GC-MS/NMR or in combination). Implementation: perform data overlap analysis, identify the differentially expressed metabolites/pathways and implement the knowledge to modify the process for achieving improved cellular performance in the bioreactor for higher yield.

Metabolomics identifies and quantifies small molecules (<1,500 Da), called metabolites, which vary in concentration based on cellular response to environmental changes and hence better reflects the performance of biological pathways and the physiologic status of a cell in certain condition/environment (Mashabela et al., 2022). Metabolomics provides unique insights into cellular metabolism and complement other “omics” sciences. The first step of metabolomics analysis is the preparation of samples (metabolite extraction from debris-free culture supernatants, washed cells or headspace of the culture vessel) followed by resolution and identification of metabolites by Liquid Chromatography (LC) or Gas Chromatography (GC) coupled with mass spectrometry (MS) or identification with nuclear magnetic resonance (NMR) (Kapoore et al., 2017) (Figure 1). Bioinformatics tools are thereafter used to connect the detected peaks (metabolites) to their identity, metabolic pathways and quantifies metabolic fluxes. The metabolomics investigations are typically of two types, untargeted and targeted. Untargeted metabolomics is an unbiased analysis measuring all detectable metabolites present in the sample (global metabolic profiling) and facilitates the discovery of new molecules impacting cell metabolism (Qiu et al., 2023). In contrast, the targeted metabolomics is a quantitative approach where a single or group of known and chemically defined metabolites (often identified using untargeted approach) are quantitated with/without ^15^N or ^13^C compounds known as labelling isotopes.

Until the last decades, our understanding of CHO cellular metabolism and metabolomics was limited to only measurement of certain nutrient metabolites (i.e., glucose and glutamine) and establish their utilization to generation and accumulation of waste metabolites (i.e., lactate and ammonia) in culture (Zhang et al., 2016). With the early success in exploring various aspects of host-pathogen interactions and disease biology, the currently available metabolomics techniques, LC-MS, GC-MS and NMR, have now started trending to elucidate cellular metabolism(s) and mechanism(s) regulating growth and productivity with a focus. However, the primary focus of studies was to identify the optimal culture media and feed components for rational media designing; but now slowly percolating towards the intercellular metabolic profiles of requisite cellular phenotypes (fast-grower and/or high-producer, clonal stability over long term culture, etc. (Coulet et al., 2022; Torres M. et al., 2023). Therefore, in this review, we have focussed on the potential of LC-MS, GC-MS and NMR for investigating the CHO metabolome during bioprocess. We have also tried to collate and discuss the published metabolite profiles of the CHO cells at different stages of bioprocess with the desired cellular phenotypes, i.e., cell growth and recombinant protein production, along with the analytical technique to detect these metabolites. We believe this knowledge may enable the development of strategies for achieving improved metabolomic coverage and generate knowledge to increase yield from the production cultures.

## 2 Currently available techniques for CHO metabolomics

**FIGURE 2:**
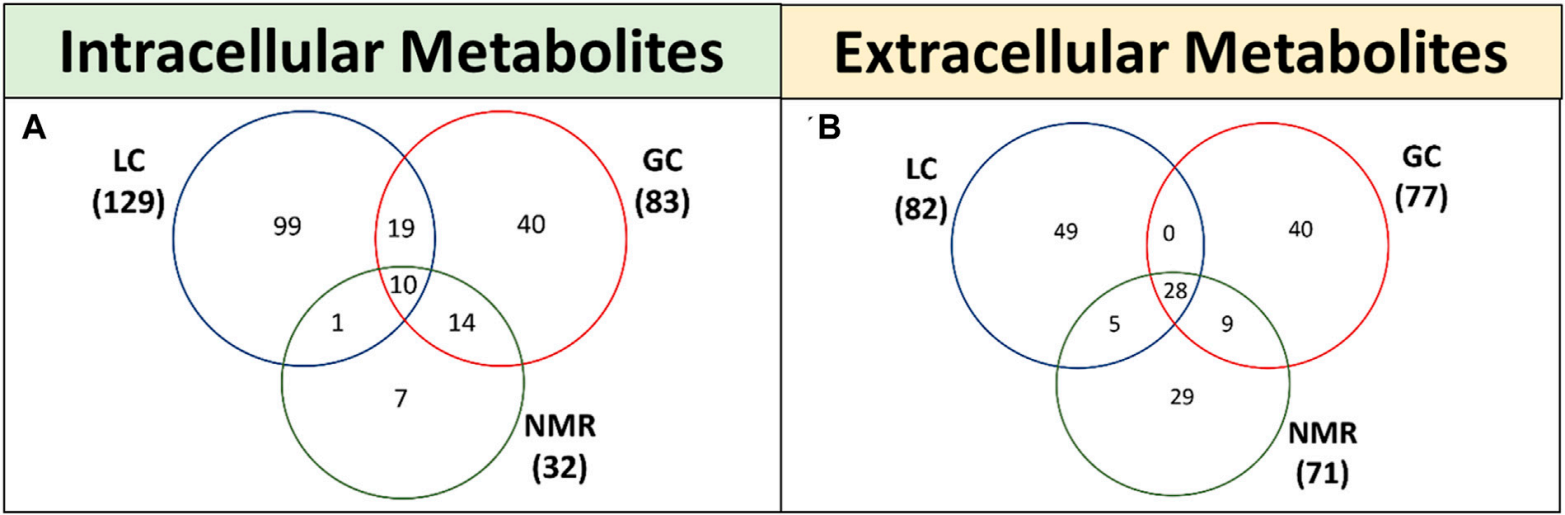
Venn diagram showing the overlap of intracellular **(A)** and extracellular **(B)** metabolites detected using LC-MS, GC–MS and NMR in different published studies. For this, the published and publicly available data sets for CHO cells were fetched and analysed.

LC-MS, GC-MS and NMR are the most commonly used techniques for CHO metabolomics. LC-MS provides highest metabolomic coverage with a wide range of metabolite classes and hence offers great potential to investigate CHO cells in culture (Figure 2; Table 1). Briefly, the liquid chromatography (LC) resolves metabolites from the mixture and mass spectrometry (MS) provides spectral information that identify (or confirm the suspected identity of) each separated component (Markley et al., 2017). MS is not only sensitive, but also provides selective detection, relieving the need for complete chromatographic separation. GC-MS can be used to study liquid, gaseous or solid samples and hence is of great importance to investigate CHO cells. Briefly, the compounds are propelled by an inert carrier gas such as helium, hydrogen or nitrogen in GC and metabolites are detected using MS (Danzi et al., 2023). On the other hand, NMR spectroscopy allows analysis of live cells, including intracellular pH and levels of phosphorylated intermediates, along with detection of novel compounds, monitor nutrient consumption and metabolite accumulation in mammalian cell cultures (Moco, 2022). Each of these techniques has its own bias towards a specific metabolite or class and technical advantages/disadvantages (Tables 1, 2). For example, out of 474 metabolites detected, 148 were uniquely detected by LC-MS, 80 were unique for GC-MS and 36 for NMR in CHO based intracellular and extracellular metabolomics studies (Figure 2). As expected, majority of the metabolites identified by LC-MS were polar, metabolites identified by GC-MS were small, volatile and non-polar molecules due to generation of robust and reproducible mass spectra from electron ionisation and metabolites identified by NMR were inorganic molecules (Supplementary Table S1). This indicates that all these techniques are complimentary to each other and hence, utilisation of multiple analytical techniques can increase the overall metabolomic coverage.

**TABLE 1:** Generic comparison of commonly used analytical techniques for investigating CHO metabolome.

Parameter		Mass-spectrometry		Nuclear magnetic resonance spectroscopy (NMR)
		Liquid chromatography–mass spectrometry (LC–MS)	Gas chromatography–mass spectrometry (GC-MS)	
General description		Samples are resolved using liquid chromatography for reducing the sample complexity and allowing metabolite separation prior to detection based on the polarity of the analytes. HILIC column is used to resolve and identify non-polar metabolites (e.g., sugars, amino sugars, amino acids, vitamins, carboxylic acids and nucleotides). C18 column is used to resolve and identify polar metabolites (e.g., phenolic acids, flavonoids, glycosylated steroids, alkaloids and other glycosylated species). The retention time of metabolites in the LC column is recorded. The resolved metabolites enter in to a mass spectrometer for recording their unique m/z ratios which enables their identification using the homology search algorithms with the databases of known metabolites	The sample is vaporized and injected onto the chromatographic column (stationary phase) with an inert gaseous mobile phase (such as helium, argon, nitrogen, carbon dioxide, and/or hydrogen). The metabolites are resolved using ramped or gradually heating based on their boiling points, pressure of the mobile phase, the chemical interactions between the metabolites in the sample and the stationary phase. The retention time of metabolites in the GC column is recorded. The eluted compounds undergo electron ionization (EI) or chemical ionization (CI), become charged and analysed with a mass spectrometer for recording their unique m/z ratios which enables their identification using the homology search algorithms with the databases of known metabolites	The sample is exposed to a magnetic field and radio frequency (rf) pulse. The nuclei of the atoms in samples absorb and re-emit this electromagnetic radiation which is detected as a signal. This emitted energy has a specific resonating frequency, which depends on several factors including the magnetic properties of the atoms’ isotopes and the strength of the magnetic field applied
Sample types	Live cells	No	No	Yes
	Intrinsic metabolites	Yes	Yes	Yes
	Extracellular metabolites	Yes	Yes	Yes
	Headspace gases	No	Yes	No
Sample preparation		The cells are mixed in ice cold metabolite extraction solvents (i.e., pure methanol, aqueous acetonitrile and/or water) and repetitive freeze-thaw cycles or sonication method is used to puncture/Lyse the cells to release and isolate metabolites	The cells are mixed with ice cold metabolite extraction solvents (i.e., pure methanol, aqueous acetonitrile and/or water) and derivatization/trimethylsilylation reagents (i.e., N,O-bis(trimethylsilyl)trifluoroacetamide and N-methyl-N-(trimethylsilyl)trifluoroacetamide) are added	It requires minimal sample preparation. Typically, the sample is transferred into an NMR tube and a deuterated locking solvent is added. No chromatographic separation, sample treatment or chemical derivatization is required
Sensitivity		High -Typically detects metabolites with mM-pM concentrations	Average- Typically detects metabolites with mM-nM concentrations	Low- Typically detects metabolites with mM-µM concentrations. Compared to LC-MS and GC-MS, NMR spectroscopy is often 10 to 100 times less sensitive
Reproducibility		Average reproducibility	Highly reproducible	Exceptionally reproducible
Number of detectable metabolites		1,000+	200–500	30–200
Sample analysis time		Average (10–30min)	High (≥30 min)	Low (1–5 min)
Cost/sample		High	High	Low
Databases		MassBank, METLIN, and mzCloud	NIST, Wiley registry, Open-access MassBank database, Golm repository	HMDB, BMRB, TOCCATA, and COLMAR
Major advantages		Highly sensitive and hence better metabolome coverage	Best suited to analyse volatile compounds	Non-destructive
			Ideal for targeted analysis	Exceptionally reproducible results
			The NIST14 library comprises GC-MS mass spectra for 242,477 unique compounds of which roughly one-third have recorded standardized retention times, enabling the use of two orthogonal parameters (mass spectral and retention index matching) for compound identification	Detection of less tractable compounds such as sugars, organic acids, alcohol, pyrols and other highly polar compounds
		Ideal for targeted analysis		Involves minimal sample preparation
				Signal intensity is directly proportional to the metabolite concentrations
Major disadvantages/limitation		Significant data variability due to variation in sample preparation, instrument condition, or operation environment which leads to drift of retention times, alteration of intensity values, and to a much less scale, drift of m/z values causing low/miss-identification output	Non-volatile compounds require derivatization. There is a possibility of unstable derivatization of amino acids, resulting in two or more peaks limiting accurate quantification and identification. A few metabolites are hard to ionize and hence may not be fit for MS analysis	Lower sensitivity (10–100 folds) compared to GC-MS or LC-MS
		Can be affected by matrix effects, varied metabolite ionization efficiency	Limited to analysing volatile and semi-volatile metabolites, some metabolites may decompose or fragment during GC separation	Not ideal for targeted analysis
		LC-MS/MS spectral libraries are significantly smaller in size, with only 8,171 unique compounds in the NIST14 library or 12,099 unique compounds in the Metlin LC-MS/MS library (which lack retention information)		NMR data bases contain only limited number of relevant compounds

**TABLE 2:** Overlapping of metabolites detected in CHO-based bioprocess using LC-MS, GC-MSand NMR for intracellular and extracellular metabolites.

Unique			Common			
LC	GC	NMR	LC and GC	GC& NMR	NMR and LC	LC, GC and NMR
Intracellular						
Total: 99	Total: 40	Total: 7	Total: 19	Total: 14	Total: 1	Total: 10
1, 2-Propanediol, 13-octadecenoate (18:1n5), 13Z, 16Z-Docosadienoic acid, 1-Methyladenosine, 2′-O-Methylguanosine, 3′-CMP, 3-methyl-2-oxobutyrate (2-Oxoisovalerate), 3-Methyl-2-oxopentanote, 4-Guanidinobutanoic acid, 4-Hydroxybutyric acid, 4-Hydroxyphenyllactic acid, 5-Hydroxymethyl-2-furancarboxaldehyde, 5′-Methylthioadenosine, 7-Dehydrocholesterol, 7-Methylguanine, Adenylsuccinic acid, Ala-Gly, alanylleucine dipeptide, Arabitol, CDP-choline, CDP-Ethanolamine, Cys-Gly, Deoxycytidine, Dimethylarginine, Erythronic acid, FAD, Fructose 6-phosphate, gamma-Glutamylalanine, gamma-Glutamylisoleucine, gamma-Glutamylleucine, gamma-Glutamylphenylalanine, gamma-Glutamylthreonine, gamma-Glutamylvaline, GDP-L-fucose, Glyceric acid, Glycero-2-phosphate, Hexanoic acid, caproate (6:0), Homocysteine, Indolelactic acid, Inosine, isoleucylglycine dipeptide, Ketoleucine, Lanosterol, Laurate (12:0), Dodecanoic acid, leucylglycine dipeptide, L-gamma-Glutamyl-cysteine, Linoleic acid, LPE 16:0/0:0, LPG 18:0, LPI 18:0/0:0, LPI 18:1/0:0, lysylleucine dipeptide, Maltulose, Mannose 6-phosphate, Methionine sulfoxide, MG 18:1/0:0/0:0, Myristoleic acid, N-Acetylalanine, N-Acetyl-D-glucosamine, N-Acetylglycine, N-Acetylisoleucine, N-Acetylleucine, N-Acetylmethionine, N-Acetylneuraminic acid, N-Acetylphenylalanine, N-Acetyltyrosine, N-Acetylvaline, NADP+, N-Formylmethionine, Nonadeca-10Z-enoic acid, Nonadecenoic acid, Norophthalmic acid, Oleic acid, Ophthalmic acid, Orotic acid, Oxalosuccinic acid, Pantetheine 4′-phosphate, Pentadecylic acid, phenylalanylglycine dipeptide, Phenyllactic acid, Phosphoethanolamine, Pro-Glu, Pro-Pro, Ribitol, Riboflavin, S-Glutathionyl-cysteine, SM 18:1; O2/16:0, Sphinganine, Sphingosine, Thymidine, trans-Vaccenic acid, Tryptamine, Tyr-Gly, UDP-Gal, UDP-galactose, UDP-glucose, UMP, valylglycine dipeptide, Xi-17-Methyloctadecanoic acid	1D-chiro-Inositol, 3-Phosphoglyceric acid, acetic acid, Adenosine, ATP, beta-Alanine, Carbonic acid, Cystine, Fructose 1, 6-bisphosphate, fructose 1, 6‐bisphosphate, fructose 2, 6‐bisphosphate, fructose 6‐phosphate, Fructose-1P, Fumaric acid, Gluconic acid, Gluconic acid-6P, glucose 6‐phosphate, Glutaric acid, Glycerol, GTP, Gulonic acid, Homoserine, hypoxanthine nucleotide, IMP, Isobutyric acid, Myo-inositol, NADPH, NH3, Palmitic acid, Palmitoleic acid, phosphatidic acid, phosphatidylcholine, phosphoric acid, Propionic acid, Sorbitol, Succinic acid, Sucrose, Threitol, Thymine, xanthine nucleotide	2-methylbutanote, Butyric acid, chloroform, ethanol, Ethanolamine, Hyrdoxy proline, Methanol	ADP, ammonia, AMP, Benzoic acid, Choline phosphate, Citric acid, Fructose, Glutathione, Glycero-3-phosphate, Glycero-3-phosphocholine, Glycine, GMP, Malic acid, NAD+, NADH, Pantothenic acid, Phosphoenolpyruvic acid, Ribose, Serine	Choline, formic acid, Histidine, Isoleucine, Isovaleric acid, Leucine, Lysine, Methionine, Phenylalanine, Proline, Pyruvic acid, Threonine, Tyrosine, Valine	Niacinamide	Alanine, Arginine, Asparagine, Aspartic acid, Cysteine, Glucose, Glutamic acid, Glutamine, Lactic acid, Tryptophan
Extracellular						
Total: 49	Total: 40	Total: 29	Total: 0	Total: 9	Total: 5	Total: 28
2-Hydroxyisocaproic acid, 2-keto-3-deoxy-6-phosphogluconate, 2-Oxoglutaric acid, 3-bisphosphoglycerate, 5-Hydroxy-tryptophan, AcetyCoA, ADP, alpha-Aminoadipic acid, AMP, Aspartate, ATP, Biotin, cis-Aconitic acid, CMP, Coenzyme A, Dihydroxyacetone phosphate, FAD, Folic acid, Fructose 1, 6-bisphosphate, Fructose 6-phosphate, gamma-Aminobutyric acid, Glucose 6-phosphate, Glucuronic acid, Glutaric acid, Glyceraldehyde 3-phosphate, Glycogen, Glyoxylic acid, GMP, Indole-3-carboxylic acid, Inosine, Isocitric acid, Methionine sulfoxide, Methylsuccinic acid, mono-methyadipate, N-Acetylputrescinium, NAD+, NADH, NADP+, nicotinamide adenine dinucleotide, nicotinamide adenine dinucleotide phosphate, Norvaline, Oxaloacetic acid, p-Aminobenzoic acid, Phosphoenolpyruvic acid, Phosphoglyceric acid, Ribulose 5-phosphate, trans-Cinnamic acid, Trigonelline, α-ketovalerate	1chiro-Inositol, 2-piperidine, Acrylic acid, Alpha- galactopyranose, ammonia, Benzoic acid, beta-Alanine, Choline phosphate, Cyclododecane, Fructose, Fructose carboxylic acid, Glucoheptulose, Glycero-3-phosphate, Glycero-3-phosphocholine, Gulonic acid, Gulose, IGF-1b, Inositol, Maltose, Mannitol, Mannopyranose, Mannose, Myo-inositol, N-Acetyglucosamine, Oleic acid, Ornithine, Palmitic acid, phosphoric acid, Phthalic acid, Propionic acid, Ribitol, Ribose, Sorbitol, Sorbopyranose, Sedoheptulose, Talose, Tetradecan, Threitol, Thymine, Xylitol, Xylose	2-Methylbutyric acid, 4-Hydroxyphenyllactic acid, 5-Adenylic acid, Adenine, Aminobutyric acid, Butyric acid, chloroform, cis-4-Hydroxyproline, Citrulline, Cytidine, Cytidine monophosphate, Deoxycytidine, ethanol, Ethanolamine, Guanosine, Hyaluronic acid, Hyrdoxy proline, inositol, Methanol, N-Acetylarginine, Niacinamide, Phenyllactic acid, Phosphoethanolamine, Poloxamer 188, Putrescine, Pyridoxine, Pyroglutamic acid, Sucrose, UMP		Acetic acid, Choline, formic acid, Fucose, Galactose, Glycerol, Isobutyric acid, Isovaleric acid, Pantothenic acid	Adenosine, Cystine, Guanine, Hypoxanthine, Uridine	Alanine, Arginine, Asparagine, Aspartic acid, Citric acid, Cysteine, Fumaric acid, Gluconic acid, Glucose, Glutamic acid, Glutamine, Glycine, Histidine, Isoleucine, Lactic acid, Leucine, Lysine, Malic acid, Methionine, Phenylalanine, Proline, Pyruvic acid, Serine, Succinic acid, Threonine, Tryptophan, Tyrosine, Valine

As of now, GC-MS has been utilized significantly in CHO bioprocessing for understanding the cellular behaviour in the production culture and to identify key metabolites and/or pathways that regulate high-growth, -survival and/or -productivity phenotypes. Whereas the other tools like LC-MS and NMR have been limitedly utilised in CHO bioprocess, thus its potential needs to be fully explored in order to get a better coverage of metabolites in CHO cells.

## 3 Metabolomics in CHO based bioprocess

One of the most important interests of the biopharma industry is high yield at lower cost (Chusainow et al., 2009; Lai et al., 2013; Tihanyi and Nyitray, 2020). Some of the key characteristics of production cell lines to protect biopharma’s interest are: monoclonality, rapid growth, roughness, stability, higher production rate and consistency in product quality (Barnes et al., 2003; O'Flaherty et al., 2020). CHO metabolism plays key role in achieving high cell-specific productivity and is typically defined as the uptake of substrates from the culture media and feeds to utilize it as carbon and nitrogen sources (Selvarasu et al., 2012; Templeton et al., 2013; Saldanha et al., 2023). However, CHO metabolism is generally believed to be inefficient and suboptimal. The nutrients at certain concentrations lead to the intra/extra-cellular accumulation of metabolites, their intermediates, and by-products as a metabolic bottleneck in key pathways and inefficient flux distribution which may increase or decrease the cell growth, productivity and protein quality (Sellick et al., 2015; Kirsch et al., 2022). A number of studies has been done on understanding CHO bioprocess using metabolomics (Table 3) with majority of them focussing on understanding the cell growth and productivity with some on cell line development, bioprocess optimisation as discussed below.

**TABLE 3:** Representative list of key metabolites/pathways identified in CHO using metabolomics-based approaches from literature.

S. No.	Analytical method	CHO cell type, media and, working volume	Expressed product	Sample preparation	Instrument used	Pathways/metabolites impacting growth	Fold change- cell density	Fold change- culture longevity	Pathways/metabolites impacting productivity and quality	Fold change- Qp	Fold change- yield	References
**1.**	LCMS	Recombinant CHO-S cells, Protein-free chemically defined medium, 4 L (fed batch)	Monoclonal IgG antibody against Rhesus D antigen	Extracellular: Culture medium filtered (cut-off-10,000 MW), centrifugation	Surveyor Plus LC system, Atlantis T3 C18 column, LC solvents- A: 0.1% formic acid in deionized water, and B: 0.1% formic acid in methanol LTQ-Orbitrap mass spectrometer (ESI mode)	Acetyl phenylalanine and dimethylarginine, tryptophan and choline	NA	NA	NA	NA	NA	Chong et al. (2009)
**2.**		CHO-mAb	mAb	Extracellular: Culture medium filtered (cut-off-10,000 MW), centrifugation	Prominence, Shimazu LC system Atlantis T3 column (4.6 × 100mm, 3um, Waters), Solvents: A being 0.1% formic acid, and B being acetonitrile 4000 QTrap, Applied Biosystems (ESI mode)	Role in apoptosis: oxidized glutathione, AMP and GMP	Fold change of VCD w.r.t. to different bioreactor at day-6 0.22	NA	NA	NA	NA	Chong et al. (2011)
**3.**		CHO-mAb, Protein free medium, 1 L shake flask	Monoclonal IgG antibody against Rhesus D antigen	Intracellular: Cell pellet lysed by ice-cold methanol + chloroform, vortexed, cold methanol/3.8 mM tricine (9:10) + chloroform, methanol–aqueous layer was collected after centrifugation and dried under vacuum pressure	ACQUITY, Waters Corporation, UPLC system with reversed phase (C18) column (ACQUITY UPLC HSS T3 Column, 2.1 × 100 mm in length, 1.7 mm particle size), Solvents—A: water, B1:methanol; and B2: acetonitrile; with 0.1% formic acid added to all the three solvents LTQ Orbitrap; Thermo Scientific mass spectrometer (ESI mode)	NA	NA	NA	Pathways: citric acid cycle, oxidative phosphorylation, glutathione metabolism, and protein glycosylation Metabolites: NADH, FAD, reduced and oxidized glutathione, activated sugar precursors	NA	NA	Selvarasu et al. (2012)
**4.**		Recombinant CHO-mAb cells, Protein-free chemically defined medium, 4 L (fed batch)	Monoclonal IgG antibody against Rhesus D antigen	Extracellular: Culture medium filtered (cut-off-10,000 MW), centrifugation	Prominence, Shimazu LC system Atlantis T3 column (4.6 × 100mm, 3um, Waters), Solvents: A being 0.1% formic acid, and B being acetonitrile	Pathway: Citric acid cycle	Fold change of VCD w.r.t. to different CHO Clone at day-6 1.3	1.9-fold improvement in integral viable cell number	NA	NA	NA	Chong et al. (2010)
					4000 QTrap, Applied Biosystems (ESI mode)	Metabolite: Malate						
**5.**		CHO GS KO parental cell line Chemically defined BMS proprietary media 5 L bioreactors, 50 mL conical tubes, and 15 mL and 250 mL bioreactors	Glutamine synthase	Supernatant samples diluted 1:10 with HPLC grade water	TripleTOF 5,600+, AB Sciex HPLC system- 1,260 Infinity, Agilent	detected 73 metabolites that significantly correlated with growth, qP, or both	Fold change of VCD, different CHO cell line w.r.t different clone at day-6 0.2	NA	metabolites in the alanine, aspartate, and glutamate metabolism pathway, and the TCA cycle showed the strongest association with qP	Fold change of qP, different CHO cell line w.r.t. different clone at day- (6-11) 0.66	1.5	Yao et al. (2021)
**6.**		CHO cell lines Chemically defined media supplemented with soy hydrolysate	mAbs	Extracellular: culture media was diluted into appropriate solvent (usually matching the initial mobile phase condition of the LC separation technique)	Agilent 1,100 or 1200SL HPLC systems Thermo-Scientific, LTQ-Orbitrap mass spectrometer with either electrospray ionization (ESI) or atmospheric-pressure chemical ionization (APCI)	NA	Fold change of VCD, different CHO cell at Day 6 0.25	NA	Ornithine, citrulline and several amino acids and organic acids correlated positively with titre	NA	NA	Kang et al. (2015)
**7.**		CHO–K1 cell line Basal medium 125 mL shake flask	IgG	Extracellular: Culture media + IS and ACN. Vortexed, centrifuged and supernatant collected for analysis	Orbitrap LC-MS system from Thermo Scientific (Waltham, MA)	growth inhibitors-GMP, ICA, NAP, HICA, MSA, CMP, TRI,ACA Negative impact of ACA on cell growth was only found in cultures where ACA was supplemented in lower concentrations	Fold change of VCD, different CHO cell at Day 7 0.30	NA	productivity inhibitors-CMP and GMP	NA	NA	Kuang et al. (2021)
**8.**		CHO-S cell line Dynamis™ AGT™ Medium, supplemented with glutamine 100 mL	IgG1 mAb	Intracellular: “Cell Culture Profiling” method package developed by Shimadzu Corporation	LCMS-8060, a triple quadrupole mass spectrometer (Shimadzu Corporation, Kyoto, Japan) coupled with a Nexera N-series UHPLC (Shimadzu Corporation, Kyoto, Japan)	several amino acids as well as pyruvic acid and pyridoxine, governed the early cell growth	Fold change of VCD,different CHO cell Test Vs. Control at Day 5 0.25	NA	TCA cycle intermediates and several vitamins highly influenced the stationary phase, in which mAb production was maximum	NA	NA	Saldanha et al. (2023)
**9.**		CHO-S cells Media supplemented with soy hydrolysates	Therapeutic proteins	Culture media was filtered using 0.45/0.1 μm membranes prior to injection	Untargeted analysis- Waters UPLC I-Class instrument connected to a Xevo G2 Q-TOF MS ESI mode Targeted analysis- Agilent 1,260 Infinity LC system coupled to an Agilent 6460 QQQ mass spectrometer using Masshunter	NA	NA	NA	potential markers of cellular productivity-Phenylalanyl-Valine, L,L-Cyclo (leucylprolyl), Tyrosyl-Leucine, N (6)-(Octanoyl)lysine, 8-Hydroxyadenine, Methyl 7-epi-12-hydroxyjasmonate, glucoside, Physoperuvine	NA	NA	Floris et al. (2019)
**10.**		CHO-mAb cell lines	mAb	NA	Agilent (Santa Clara, CA) 6890 GC connected to an Agilent 5973 M ESI mode	Glutamine and Asparagine Fuel the TCA Cycle During Exponential Growth Phase	Fold change of VCD of HP vs. LP at Day 6 0.769	NA	Glucose, glutamine and pyruvate	NA	0.142	Dean and Reddy (2013)
**11.**		CHO-mAb 5L glass bioreactors Iscove’s Modified Dulbecco’s Medium (IMDM) and MCDB medium	mAb IgG	Extracellular: samples thawed on ice and mixed with pure methanol (1:3 v/v), vortexed, centrifuged, supernatant collected and dried using a SpeedVac concentrator The dried sample reconstituted in one-half sample volume of methanol/water (1:1 v/v)	AB SCIEX TripleTOF 5,600+, Framingham, MA, USA	Tryptophan Metabolism Negatively Correlates with Growth	Fold change of VCD of differentCHO cell3 Vs. Cell line 4 at Day 7 0.58	NA	NA	NA	NA	Alden et al. (2020)
**12.**		CHO-mAb 10 L, 100 L, and 1,000 L	mAb	intracellular samples	NA	Intracellular metabolic profiles of the CHO fed‐batch culture were shown to be consistent with scale	Fold change of VCD at different cultivation scale of CHO cell at Day 6 0.32	NA	D-glucose, L-asparagine, L-glutamine	NA	NA	Vodopivec et al. (2019)
**13.**	LC-MS	CHO host cell, animal material-free in-house growth (or base) and feed media supplemented with indicated iron compounds or with commercial iron supplements, 2 L	mAb	samples were loaded by autosampler onto a C18 trap column (0.3 50 mm, Dionex, Sunnyvale, CA) with 5% solvent B (0.1% formic acid in 97% ACN) at 10 lL/min for 5 min. Then, the peptides were separated by a nanobore picofrit column (C18, 75 lm 150 mm, 100 A˚, New Objective, Woburn, MA) using a 120 min gradient from 5% to 95% B at a flow rate of 350 nL/min, where solvent A was 0.1% formic acid with 3% ACN in HPLC grade water	Nano LC-ESI MS/MS in data-dependent acquisition mode with nano 2D HPLC system (Ekisigent, Dublin, CA) and LTQOrbitrap mass spectrometer (Thermo, Waltham, MA) equipped with a nanoelectrospray ion source (Picoview PV500, New Objective, Woburn, MA)	Sodium Citrate, ferrous ammonium citrate, ATP, 20% higher levels of glucose and lactate	NA	NA	a-ketoglutarate, fumarate, oxaloacetate, isocitrate, malate, succinate, and pyruvate showed the strongest association with qP	35%–40% enhancement of mAb production titer	NA	Bai et al. (2011)
**14.**	LC-MS	CHO DP-12, TC-42 medium, 4 mM L-glutamine and 200 nM Mtx 3.5 L	IgG	Whole cell and subcellular samples were extracted by adding 5 mL ice-cold 70% (v/v) methanol and incubated in a cryostat at −20 °C for 90 min Collected permeates were merged with 0.5 mL of ice-cold chloroform and mixed by vortexing (20 s) Resulting solutions were centrifuged at 3,200 *g* at 0 °C for 10 min upper aqueous phases (metabolite extracts) were aliquoted (1 mL) in 1.5 mL and evaporated for 110 min at 4 °C and stored at −70 °C	Agilent 1200 HPLC system coupled with an Agilent 6410B triple quadrupole mass spectrometer with an electrospray ion source	Malate and pyruvate as intermediary electron carriers	NA	NA	NA	NA	NA	Wijaya and Takors (2021)
**15.**	GCMS	Suspension adapted CHO-K1 cell line Chemically-defined medium supplemented with cottonseed hydrolysate	IgG	Extracellular: Culture media centrifuged, supernatant collected and analysed	YSI 2950 analyzer and GC-MS (Agilent 7890 GC with 5977 MSD	Reduced glycolytic pathway (glucose, lactate, galactose) possibly due to reduced GAPDH Fold change in metabolites-Glucose ∼ −1 (at t = 9 h) Lactate ∼ −0.8 (at t = 9 h) Galactose∼ 9 (at t = 9 h)	∼2 (at t = 9 h)	∼0.6 (at t = 9 h)	Processes related to “protein production” including RNA processing, translation, ribosomal assembly as well as protein folding, glycosylation, and transport were found to be enriched	0.75 (at t = 9 h)	1.5 (at t = 9 h)	Ladiwala et al. (2023)
**16.**		CHO-KI cells RPMI 1640 medium and Ham’s F12 15 mL	IGF1	Extracellular: Metabolite pellets resuspended in methoxyamine hydrochloride in pyridine (40 mg/mL) MSTFA +1% TMCS (90 ul) added and incubated. Samples cooled to RT and transferred into silanized GC vials	7890A GC System (Agilent Technologies, Santa Clara, CA, USA) coupled to a 5975C Inert XL MSD	Asparagine was found to be indicative of healthiness of cells and production of high IGF-1. Ornithine and lysine associated with apoptosis Fold change in metabolites in RPMI 1640 culture w.r.t. Ham’s F12- Glucose ∼ −1 (t = 64 h) Lactate ∼ −0.5 (t = 80 h) Glutamine∼ 2.75 (t = 48 h)	Fold change due to RPMI 1640 = −0.33 (w.r.t. Ham’s F12 at t = 32 h) and 0.66 (w.r.t. Ham’s F12 at t = 80 h)	NA	NA	NA	Fold change due to RPMI 1640 = 0.03 (w.r.t. Ham’s F12 at t = 64 h)	Mohmad-Saberi et al. (2013)
**17.**		Adherent and serum dependent CHO cell line HyQ SFM4CHO media 25 mL	human growth hormone	Intracellular: Cell suspension + ice- cold 0.9% (w/v) NaCl solution, centrifuged twice and pellet resuspended in ice-cold NaCl solution firstly and in 50% aq. ACN secondly. Extraction by 40 nmol ribitol and 250 nmol norvaline per sample. After extraction, samples spun and the supernatant collected. Metabolite extracts were freeze dried	GC- 7890A, Agilent Technologies MS- 5975C, Agilent Technologies, Mulgrave, Australia) in EI mode	Metabolites correlated with growth- dCTP, CTP, ATP, GTP, NAD, UDP-glucuronic acid (UDP-glcA), thymine, glutamine, glutamate, asparagine, histidine, glycine	Fold change due to SFM media = −0.07 (w.r.t. CDCHO at t = 120 h) Fold change due to HyQ media = 0.3 (w.r.t. CDCHO at t = 120 h)	Fold change due to SFM media = −0.1 (w.r.t. CDCHO at t = 120 h) Fold change due to HyQ media = −0.01 (w.r.t. CDCHO at t = 120 h)	NA	NA	NA	Dietmair et al. (2012)
**18.**		Suspension‐adapted CHO‐K1 cell line AMBIC 1.0 References community basal medium) 125 mL shake flask	IgG	Intracellular: Cell culture media + IS solution, water, and ACN with 1% FA mixed, vortexed, sup collected after centrifugation for analysis	Thermo Scientific Ultimate 3000 HPLC system; SeQuant ZIC‐cHILIC column coupled with a Thermo Scientific TSQ Quantiva triple quadrupole mass spectrometer	Identified 5 AA targets, Lys, Ile, Trp, Leu, Arg, as key contributors to inhibitory metabolites (CMP, NAP, ACA, TRI, HICA, and MSA) ACA accumulation- 3-fold increase in 2x‐AA and 15-fold increase in 3x‐AA condition	NA	NA	NA	NA	NA	Ladiwala et al. (2023)
**19.**	GC-MS	CHO-K1 serum free CDCHO (with 5 mM asparagine, 5 mM serine, 50 mM glucose and 25 mM MSX), 25 mL	IgG	Intracellular: Quenching with ice-cold 0.9% (v/v) NaCl solution +1min centrifugation at 1,000g and extraction 4C ice-cold 50% acetonitrile/water	QP2010 mass spectrometer (Shimadzu, Japan) in a EI mode (70 eV)	Asparagine and serine	NA	NA	NA	NA	NA	Duarte et al. (2014)
**20.**	GC–MS	CHO-K1 cells, Dulbecco’s modified Eagle medium (DMEM, Cat. No. 10–013-CV) supplemented with 10% fetal bovine serum (FBS, Cat. No. 35–011-CV) and 1% penicillin–streptomycin (PS)	NA	Extracellular: vortexed vigorously for 1 min and phase separation using centrifuged at 2000 rpm and 4 °C for 20 min	Agilent 7890A GC	Glycolysis, pentose phosphate pathway, TCA cycle, anaplerotic and cataplerotic reactions, amino acid metabolism, lactate metabolism, fatty acid metabolism	NA	NA	NA	NA	NA	Ahn and Antoniewicz (2011)
**21.**	NMR	CHOK1 and CHO-S DMEM + L-glutamine, glutamic acid, asparagine, adenosine, guanosine, cytidine, uridine, thymidine, non-essential amino acids dialyzed foetal bovine serum 10 mL	NA	Extracellular: media was combined with D2O, DSS and 5% sodium azide Dried media metabolites and quenched cell extracts were resuspended in D2O, DSS and sodium azide	UnityINOVA 600 MHz NMR spectrometer	Growth-limiting metabolites identified- alanine, lactate FC(E) alanine and lactate CHO-K1: TS to 10°C = 0.29 and 0.06 (w.r.t. 37°C at t = 216 h) FC(I) alanine and lactate CHO-K1: TS to 10°C = ∼ −0.47 and ∼0 w.r.t. 37°C at t = 144 h) FC(E) alanine and lactate CHO-S: TS to 10°C = 0.29 and 0.33 (w.r.t. 37°C at t = 216 h)	NA	NA	NA	NA	NA	Wagstaff et al. (2013)
**22.**		CHO-S cell lines 100 mL	Recombinant antibody	Extracellular: Spent medium filtered	Varian four-channel VNMRS 700 MHz NMR spectrometer	Growth inhibitory effect: 3-phenyllactate and 4-hydroxyphenyllactate, isovalerate, isobutyrate and 2-methylbutyrate	Fold change due to 4 mM 2-methylbutyrate at day 6 = −0.66 Fold change due to 4 mM Isobutyrate at day 6 = −0.67	NA	NA	NA	NA	Mulukutla et al. (2019)
**23.**		CHO-S cells 250 mL	Antibody	Extracellular: Centrifuged spent medium + D2O solution +30 mM phosphate buffer	Varian 600 MHz NMR	citric acid cycle, amino acid degradation, glycerolipid metabolism, and glycolysis pathways, acetate, formate	NA	NA	histidine	NA	0.44 (t = day 10)	Bradley et al. (2010)
**24.**		CHO-S cells media with and without dextran sulfate 7–5000 L	recombinant fusion protein	Extracellular: culture media supernatants +0.2 mL of 99.9% D2O+ 0.1 mM TSP	NMR Bruker spectrometer (Bruker Analytik, Rheinstetten, Germany)	Amino acids, Krebs cycle intermediates, activated sugars, cofactors Citrate −3.1 Acetate 23.6 Niacinamide 39.1 Uracil 64.3 Glucose 17.5 Galactose 12.5	NA	NA	NA	NA	NA	Aranibar et al. (2011)

### 3.1 Growth-associated metabolites

**FIGURE 3:**
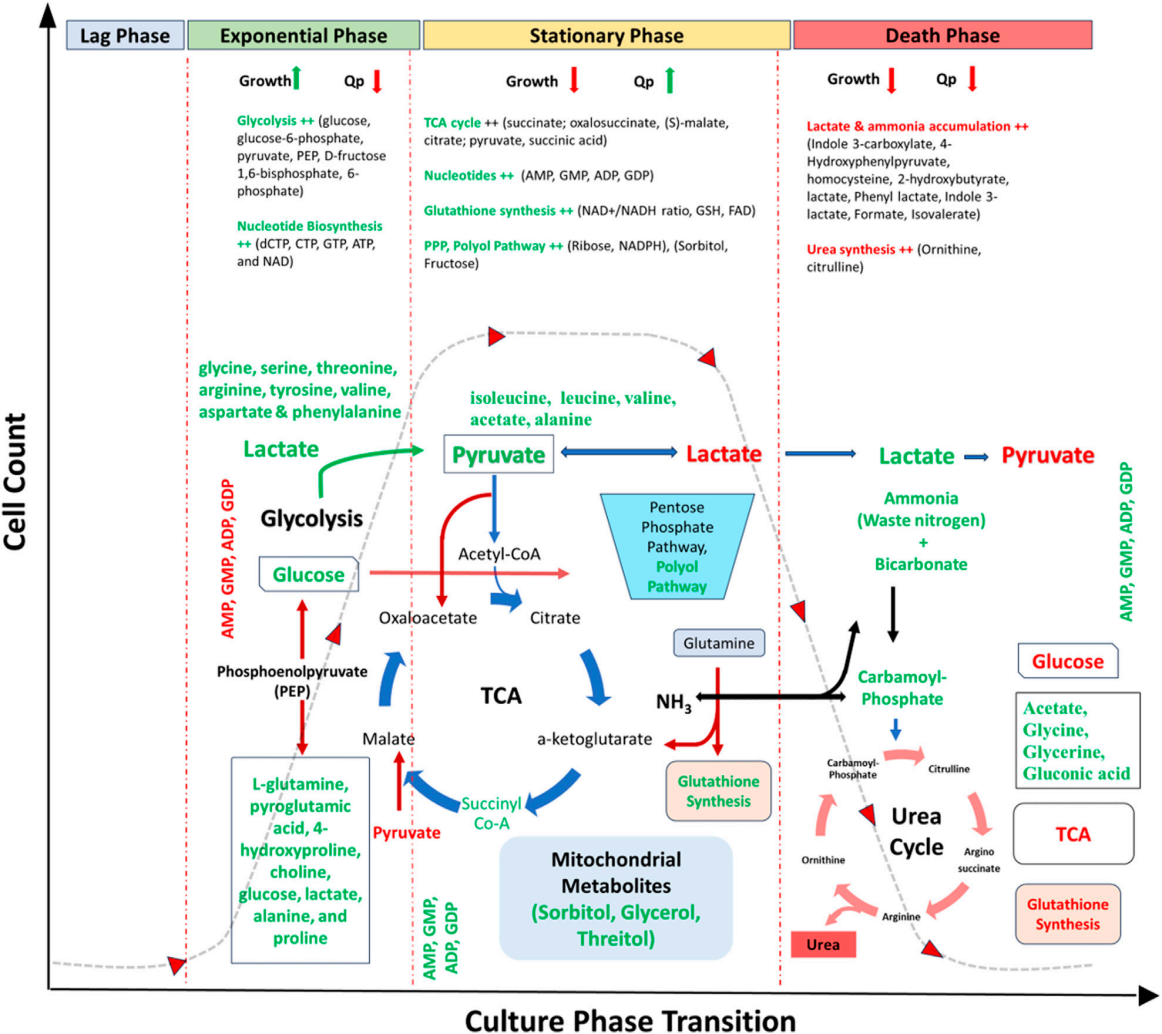
Metabolic overview of CHO culture representing key metabolites and pathways expressed over different phases of cell culture. Red represents downregulated metabolites/pathways, and green represents upregulated metabolites/pathways. Abbreviations: TCA- Tricarboxylic acid cycle, PPP- Pentose Phosphate Pathway, dNTPs-deoxy Nucleoside Triphosphates, Qp-cell specific productivity, AMP- Adenosine monophosphate, GMP- Guanosine monophosphate, ADP- Adenosine diphosphate, GDP- Guanosine diphosphate, dCTP- Deoxycytidine triphosphate, CTP- Cytidine triphosphate, GTP- Guanosine triphosphate, ATP- Adenosine triphosphate, NAD-Nicotinamide adenine dinucleotide, NADH- nicotinamide adenine dinucleotide (NAD) + hydrogen, GSH- Glutathione, FAD-flavin adenine dinucleotide.

The glycolytic pathway and its associated pathways upregulates in the exponential phase of culture to potentially meet the increased demand of energy during cell proliferation (Sengupta et al., 2011; Templeton et al., 2013; Zhu et al., 2022; Naik et al., 2023) (Table 3; Figure 3). For example, glucose, glucose-6-phosphate, pyruvate, phosphoenolpyruvate, fructose 1,6-bisphosphate, and fructose 6-phosphate increases in the exponentially growing cells (Buchsteiner et al., 2018; Zhang et al., 2021; Coulet et al., 2022). Majority of these metabolites can be detected using GC-MS, except glucose which can be universally identified using any of the three techniques (LC-MS, GC-MS, NMR) (Supplementary Table S1). These metabolites are highly utilised in the exponential phase of cells thereby increasing the cell proliferation (Hsu et al., 2017). The metabolites of gluconeogenesis pathway (L-glutamine, UMP, deoxycytidine, orotate, acetate, glycerone phosphate) also upregulate to support the increasing demand of energy during this growth phase (Supplementary Table S1). Besides the intracellular level of UDP-glucose, detected using LC-MS, was also reported increased in growing cultures with low level of UDP-glcA due to reduced activity of UDP-glucose 6-dehydrogenase, however both are crucial for cell growth. Therefore, improving the levels of UDP-glucose 6-dehydrogenase by improved media or process design and cell engineering approaches may overcome the bottleneck of cellular metabolism for growth (Dietmair et al., 2012).

The TCA cycle metabolic fluxes (succinate; oxalosuccinate, (S)-malate, citrate; pyruvate, succinic acid) remains upregulated at the stationary phase as compared to the exponential phase (Bai et al., 2011; Sengupta et al., 2011; Dietmair et al., 2012; Templeton et al., 2013; Duarte et al., 2014). Most of these metabolites were detected by both LC-MS and NMR, except fumarate and succinate which were reported to be only detected using GC-MS (Supplementary Table S1). This indicates that the cells at stationary phase mostly rely on oxidative phosphorylation pathway for ATP generation, whereas the cells at exponential phase primarily depends on glycolysis for ATP production via substrate level phosphorylation. As the growth rate decreases (stationary-phase), the metabolic-shift to utilize glucose in the TCA cycle increases to achieve high viable cell mass and antibody titres (Dean and Reddy, 2013). A significant portion of the pyruvate formed from the glucose is converted into lactate, which is secreted and acidifies the medium, with the rest being used to supply the TCA cycle thus channelising the cells into the stationary phase indicating the shift from lactate producers (glucose consumers) to lactate consumer cells (Selvarasu et al., 2012; Wijaya et al., 2021). However, contrary to glucose consumption, glutamine is also reported to be utilized more efficiently than glucose for anaplerotic replenishment of TCA intermediates and contributed more significantly to lactate production during the exponential phase (Templeton et al., 2013). Therefore, optimizing the concentration of glycolytic and TCA intermediates, (ornithine, pyridoxine and pyruvic acid in the growth medium, and citric acid and succinic acid in the feed medium) can help to regulate cell growth in culture (Saldanha et al., 2023). The significant flux of oxidative pentose phosphate pathway (oxPPP) has also been reported to be upregulated indicating additional requirement of NADPH and glutathione, which contributes in fighting oxidative stress during the stationary phase (Ahn and Antoniewicz, 2011; Sengupta et al., 2011). PPP metabolites were mostly detected using LC and GC-MS only (Supplementary Table S1). Therefore, stationary phase may be characterized by a reduced glycolysis flux, lactate uptake, low consumption of carbon and nitrogen sources, increased oxPPP flux, and reduced rate of anaplerosis.

All the 20 amino acids can be detected using all three techniques (LC, GC, NMR) in different phases of CHO cell culture; however, most of them are uniformly detected by GC-MS, followed by NMR (Supplementary Table S1). Intracellular level of a number of amino acids (glycine, serine, threonine, arginine, tyrosine, valine, aspartate and phenylalanine) are significantly lower in the stationary phase compared to the exponential phase (Lu et al., 2005; Kirsch et al., 2022). Depletion of specific amino acids, including arginine, cysteine, histidine, phenylalanine, tryptophan and pyruvate, leads to the initiation of the decline phase in the CHO cultures (Sellick et al., 2015). The depletion of eight metabolites, including glucose, glutamine, proline, serine, cystine, asparagine, choline, and hypoxanthine, from the production media of stably antibody-expressing CHO cells exerts cellular stress limiting the cell growth and supplementation of these metabolites as a nutrient cocktail result in improved peak cell density by ∼ 75% (Sellick et al., 2015). These amino acids are known to enter into catabolic processes thereby leading to their high consumption in culture, to complement the energy gain from glucose (Fan et al., 2015). Thus, decreased level of many of these amino acids in stationary phase of culture may potentially be indicating the exhaustion of other possible substrates and their utilisation for energy generation, instead of being used as building blocks in protein synthesis. However, contradictorily cysteine is reported to induce senescence and deaccelerate cell growth (Chu and Liu, 2015). Moreover, 5-hydroxy indole acetaldehyde (5-HIAAld), a tryptophan metabolite, has a strong negative correlation with peak viable cell density and hence tryptophan supplementation is also observed to have growth-inhibitory effects potentially leading to excessive accumulation of 5-HIAAld in the culture medium (Alden et al., 2020).

Nucleotides (dCTP, CTP, GTP, ATP, and NAD) also play an important role in cellular metabolism in the growth phase, acting as building blocks for the synthesis of RNA or DNA and/or cofactors in a large number of reactions (Dietmair et al., 2012). These nucleotides can be mostly detected by both LC and GC-MS (Supplementary Table S1). Contrastly, few nucleotides (ATP, AMP, GMP, ADP, GDP, adenosine) are predicted to induce growth arrest of CHO cells in the G1 phase thereby channelising them from exponential to stationary growth phase (Pereira et al., 2018). Additionally, various vitamins (choline chloride, i-inositol, niacinamide, folic acid, thiamine, pyridoxine) and hormones (choline chloride, triiodothyronine (T3) and human insulin like growth factor-I (IGF-I) enhances the culture performances through increased growth and productivity (Kim et al., 2005; Selvarasu et al., 2012).

Besides, the waste products accumulated in production culture due to inefficient metabolism also impacts the growth and productivity. Consumption of glutamine and asparagine leads to generation and accumulation of ammonia in culture which are well known to negatively affect the cell-growth, product quality and productivity (Selvarasu et al., 2012). Reduced accumulation of other growth-limiting waste metabolites (lactate and alanine) is also associated with increased culture longevity and protein productivity in hypothermia-based biphasic cultures (Wagstaff et al., 2013). Similarly, indole 3-carboxylate, 4-hydroxyphenylpyruvate, homocysteine, 2-hydroxybutyrate, lactate, Phenyl lactate, Indole 3-lactate, Formate, Isovalerate (intermediates or byproducts) of amino acid catabolism also inhibits the cell growth in fed-batch cultures of Chinese hamster ovary cells (Mulukutla et al., 2017). Controlled supplementation of some of these amino acids during the growth phase can reduce the rate of their production and accumulation in later-stage culture, improving peak-cell density and productivity from culture (Mulukutla et al., 2017). For example, reduced generation and accumulation of three branched-chain amino acids (isovalerate, isobutyrate and 2-methylbutyrate) by knocking-out the gene branched-chain amino acid transaminase 1 (BCAT1) coding an enzyme required for their production helped in retaining high culture viability (≥95%) even on the 20th day of fed-batch culture (Mulukutla et al., 2019). Minimizing the accumulation of intermediates and byproducts of the phenylalanine-tyrosine pathway by over-expressing the genes involved in their effective conversion enabled the culture to achieve higher peak-cell density and productivity (Coulet et al., 2022). Recently, the extracellular detection of L-glutamine, pyroglutamic acid, 4-hydroxyproline, choline, glucose, lactate, alanine, and proline were proposed to be the biochemical markers of the logarithmic growth phase whereas isoleucine, leucine, valine, acetate, and alanine for the stationary growth phase and acetate, glycine, glycerine, and gluconic acid for the cell decline phase.

Therefore, metabolic engineering for efficient characterization of nutrients for growth and protein production and to minimize generation and accumulation of growth limiting metabolites in culture could help to improve the cell growth and productivity (Zhao et al., 2023).

### 3.2 Productivity-associated metabolites

Metabolites in the TCA cycle and its intermediate/precursors (alanine, aspartate, and glutamate metabolism pathway), PPP, glutathione synthesis pathway and polyol pathway have the strongest association with qP (Dietmair et al., 2012) (Table 3; Figure 3). Significantly upregulated TCA cycle (including citric acid, isocitric acid, α-ketoglutarate and glutamate) and downregulated intracellular glycolytic pathway (*i.e.*, 3-phosphoglycerate) with a metabolic-shift from lactate accumulation to consumption and lipid metabolic pathways (i.e., choline and phosphoryl-choline that is required for membrane phospholipids) are associated with reduced growth and increased cell-specific and overall productivity (Zhu et al., 2022).

Increased intracellular levels of three activated sugar precursors (GDP-fucose, UDP-Gal/UDP-Glc, and UDP-GlcNAc) potentially associated with protein glycosylation, have been reported in CHO cells with increased qP for mAbs (Chong et al., 2012). These sugar precursors can be majorly detected using GC-MS except few (UDP-Gal, UDP-galactose, UDP-glucose) which were uniquely identified in studies performed using GC-MS, whereas NMR was unable to detect these polar metabolites (Supplementary Table S1). An efficient glycometabolism for protein production allows less glucose uptake by cells (Zhang et al., 2021). Besides, reduction of lactate levels in CHO cell cultures improves the product titre by 11%–32% without having significant impact on cell growth suggesting that reduced level of lactate in culture potentially either due to its consumption or inhibited generation may be the primary cause of its beneficial effects (Ahn and Antoniewicz, 2011; Naik et al., 2023).

Enhanced TCA activity is the predominant distinguishing feature between high and low-producer CHO cell lines (Saldanha et al., 2023). Supplementation of TCA intermediates, or their precursors (aspartate, glutamate, succinic acid, malic acid, fumaric acid) in the stationary phase of culture increases cell growth and mAb production by > 50% without affecting its quality (Saldanha et al., 2023). These TCA cycle intermediates can be mostly detected using LC-MS except fumaric acid which is observed to be uniquely detected by GC-MS (Supplementary Table S1). Moreover, supplementation of TCA intermediate, citrate, alone was observed to increase the qP by up to 490% and more than doubled the titre (Yao et al., 2021); this might be because of the amount of substrate available for the TCA cycle is increased and further, citrate also promote iron chelation that ultimately led to increased qP (Bai et al., 2011; Zhang et al., 2020). Similarly, aspartate was also found to increase the recombinant protein production (Yao et al., 2021). Besides, intracellular and extracellular levels of mitochondrial metabolites (sorbitol, glycerol, threitol) and polyol pathway (high glucose, fructose, sorbitol) are increased in cells with higher cell-specific productivity indicating the cellular need for increased citric acid cycle function and mitochondrial oxidative capacity to achieve higher productivity and is a characteristic for high-producing CHO cell lines (Templeton et al., 2013; Sellick et al., 2015; Templeton et al., 2017).

Addition of a few amino acids (glycine, methionine, phenylalanine, threonine, tyrosine) also increases the productivity of CHO cells by inhibiting their growth. Their accumulation in the culture medium during the growth phase mediates the transition of exponential to stationary phase. These metabolites inhibit the pyruvate kinase which produces ammonia through pyruvate transamination during late stages of culture (Wijaya et al., 2021; Pereira et al., 2018). The amino acids, glutamine and asparagine present inside the cell milieu has often been correlated with the productivity and healthiness of the cells in culture (Wishart et al., 2009; Mohmad-Saberi et al., 2013). However, increased glutamine supplementation also elevates lactate, alanine, and ammonia fluxes during the early exponential phase, which is known to impact growth and productivity in cultures through TCA cycle (Kirsch et al., 2022). Therefore, a balanced supplementation of glutamine in the media is recommended to support the elevated level of glutathione, nucleotides and nucleotide sugars (such as UDP‐GlcNAc) that has been associated with increased productivity. Depletion of histidine, which is involved in folding and assembly of newly synthesised proteins, from the culture media was observed to significantly decrease the production of recombinant antibody without significantly impacting the CHO cell growth and hence increased availability of histidine in the culture medium may improve the productivity (Lu et al., 2006; Ladiwala et al., 2023). Contrarily reduction of growth and productivity inhibitory metabolites (Lysine, Isoleucine, Tryptophan, Leucine and Arginine) in medium decreased the accumulation of inhibitory metabolites and improved growth and IgG production in the batch and fed-batch processes (Ladiwala et al., 2023). Similarly, production and accumulation of eight metabolic derivatives including aconitic acid, 2-hydroxyisocaproic acid, methyl succinic acid, cytidine monophosphate, trigonelline, and n-acetyl putrescine, in culture media due to inefficient cell metabolism were reported to reduce cell growth (∼27%) and productivity (∼40%) besides inhibiting the formation of mono-galactosylated biantennary (G1F) and biantennary galactosylated (G2F) N-glycans of the antibody (Kuang et al., 2021). Further, these accumulating metabolites are product associated with branched-chain amino acids, tryptophan, nicotinamide and polyamine pathways (Harrington et al., 2021).

Intracellular NAD+/NADH ratio, intracellular NADPH, FAD and glutathione metabolic pathways are also upregulated which might be reasoned to the fact that NADPH is a vital cofactor and crucial redox partner in various cellular reactions and contribute to anabolic reactions including pathways of citric acid cycle, oxidative phosphorylation, glutathione metabolism, and protein glycosylation (Chong et al., 2012; Hosios and Vander Heiden, 2018) affecting the overall qP. Besides, the pentose phosphate pathway is also upregulated, potentially fulfilling the increased demand of energy (NADPH) of cells with qP (Buchsteiner et al., 2018).

Taken together from LC-MS, GC-MS and NMR-based metabolomics, the higher availability of metabolites involved in TCA cycle and amino acids are vital to support the cell growth while the upregulation of metabolites of the TCA cycle, polyol pathway, mitochondrial oxidative capacity and glutathione metabolism during the stationary phase of culture are important for both, cell growth and productivity, in CHO cell culture.

### 3.3 Cell line development and process design

Recently, distinct metabolomic signatures (increased consumption of glucose, amino acids, accumulation of greater amounts of lactate and TCA cycle intermediates) were identified for long-term cultured (long-passage) cells which typically adapt metabolome towards cell proliferation and survival instead for productivity (product yield and quality) compared early-passaged cells (Torres and Dickson, 2022; Kaur et al., 2023). Metabolic characterization of CHO cells also led to identification of an additional phase called the cell size increase (SI) phase that occurs between the exponential proliferation phase (also called number increase (NI) phase) and the stationary phase, during which the cell division comes to a halt but the cell growth continues in the form of an increase in cell size, thereby increasing average volume and dry weight per cell by threefold with time (Pan et al., 2017). The average mAb specific productivity per cell increases linearly with the cell volume curating two times higher in the SI phase than NI phase. Accumulation of fatty acids and formation of lipid droplets in the cells are observed during the SI phase, indicating that the fatty acids synthesis rate exceeds the demand for the synthesis of membrane lipids (Pan et al., 2017). The CHO cells showed increased under hydrostatic pressure (≥60 mmHg) cell-specific glucose consumption rate, cell-specific lactate production rate and cell-specific ammonium production rate under hydrostatic pressure (≥60 mmHg) compared to 0 mmHg. However, an increase cell proliferation and productivity observed under ∼30 mmHg (Shang et al., 2021). Besides, metabolomics of different CHO cell line (CHO-K1, CHO-GS, CHO-S and CHO-DG44) reflect a clear heterogeneity among them, with each having some unique metabolites being detected (Supplementary Table S1), which may be either due to the difference in their culture media used during investigation, expressing different product and/or process design. More interestingly, no intracellular metabolite has been observed to be significantly differentially expressed to connect the scale‐up effect among 10L, 100L and 1,000L bioreactor cultures (Vodopivec et al., 2019).

## 4 Gaps of metabolomic pipeline

Metabolomics, as of now, has primarily been utilized to evaluate the effects of growth medium composition on cell growth and productivity and hence, facilitated rational medium design leading to significant improvements in CHO-based bioprocessing and thereof yield. However, there is much more to be explored by incorporating the recent advancement in current metabolomic tools for extracellular as well as intracellular metabolite analysis which may not be feasible without fixing the currently existing technical gaps in the metabolomics pipeline as detailed below-

### 4.1 Biasness of analytical technique(s)

Each metabolomic technique has its own biasness towards specific metabolites or class of metabolites depending upon their physicochemical properties (polar, lipids, organic acid, etc.) (Table 2). For instance, LC-MS is more suited for the identification of large, polar, ionic, or non-volatile, thermally unstable metabolites (e.g., most amino acids, NAD, NAG, FAD, etc.), GC-MS is more suited to volatile organic compounds, short-chain fatty acids, sugars and hydroxyl acids (e.g., putrescine, tetradecane, 2-hydroxycaproic acid, etc.) (Supplementary Table S1). And, NMR is preferred when sample treatment or chemical derivatization is not desired and to detect specific inorganic metabolites/ions and protein-bound metabolites which are not detected or distinguishable by MS techniques (e.g., leucine, isoleucine, etc (Behera et al., 2020).

### 4.2 Low populated databases and metabolomic coverage

Although, LC-MS-based approaches detect the highest number of metabolites (>4000 features) in a cell and have been proven the most feasible approach for omics-based studies, but its spectral libraries are smaller in size (8,171 compounds in NIST14 library, 12,099 compounds in Metlin library, lacking retention information). Whereas, the NIST14 library for GC-MS comprises mass spectra for 242,477 unique compounds, of which roughly one-third have records for standardized retention times. An ultra-high performance metabolomics platform can detect up to 7,000 metabolic features in a typical application (Dunn et al., 2011). However, the majority of the studies as of now have been only able to identify a lesser number (∼350 metabolites), suggesting that the analytical coverage of a metabolome is far from achieving the expected range and thereby might miss the critical metabolites that actually regulate cell growth and productivity in culture.

### 4.3 Biasness of metabolite extraction methods

Different metabolite quenching and extraction methods have biased towards different classes of compounds (amino acids, carbohydrates, nucleotides, sugars, etc.). Hence, cataloguing methods with respect to their biasness towards metabolite extraction for general use can facilitate the detection of a larger range of metabolites or help in the quantification of a specific/targeted metabolite(s) (Kumar et al., 2022; Singh et al., 2023). For example, the extraction of metabolites using 100% methanol extraction followed by water was found to be most effective for the recovery of the largest range of metabolites in CHO cells by GC-MS (Sellick et al., 2010). Derivatisation with propyl chloroformate/propanol obtains excellent extraction of amino acid analytes (Batista et al., 2023). The recovery of fatty acids (e.g., stearic acid and palmitic acid) was maximized by hot ethanol extraction, while the recovery of glycerol-1-phosphate was significantly greater when cellular metabolites were extracted with KOH (Park et al., 2021). On the other hand, polar metabolites are extracted with cold methanol + MSTFA (N-Methyl-N-trifluoroacetamide) + 1% TMCS (trimethylchlorosilane); organic acids, ketoacids by BSTFA (N,O-Bis(trimethylsilyl)trifluoroacetamide) and bile acids by methanol +2% sulfuric acid + MSTFA +1% TMCS for NMR (Bouatra et al., 2013).

### 4.4 Metabolomic variability among hosts

**FIGURE 4:**
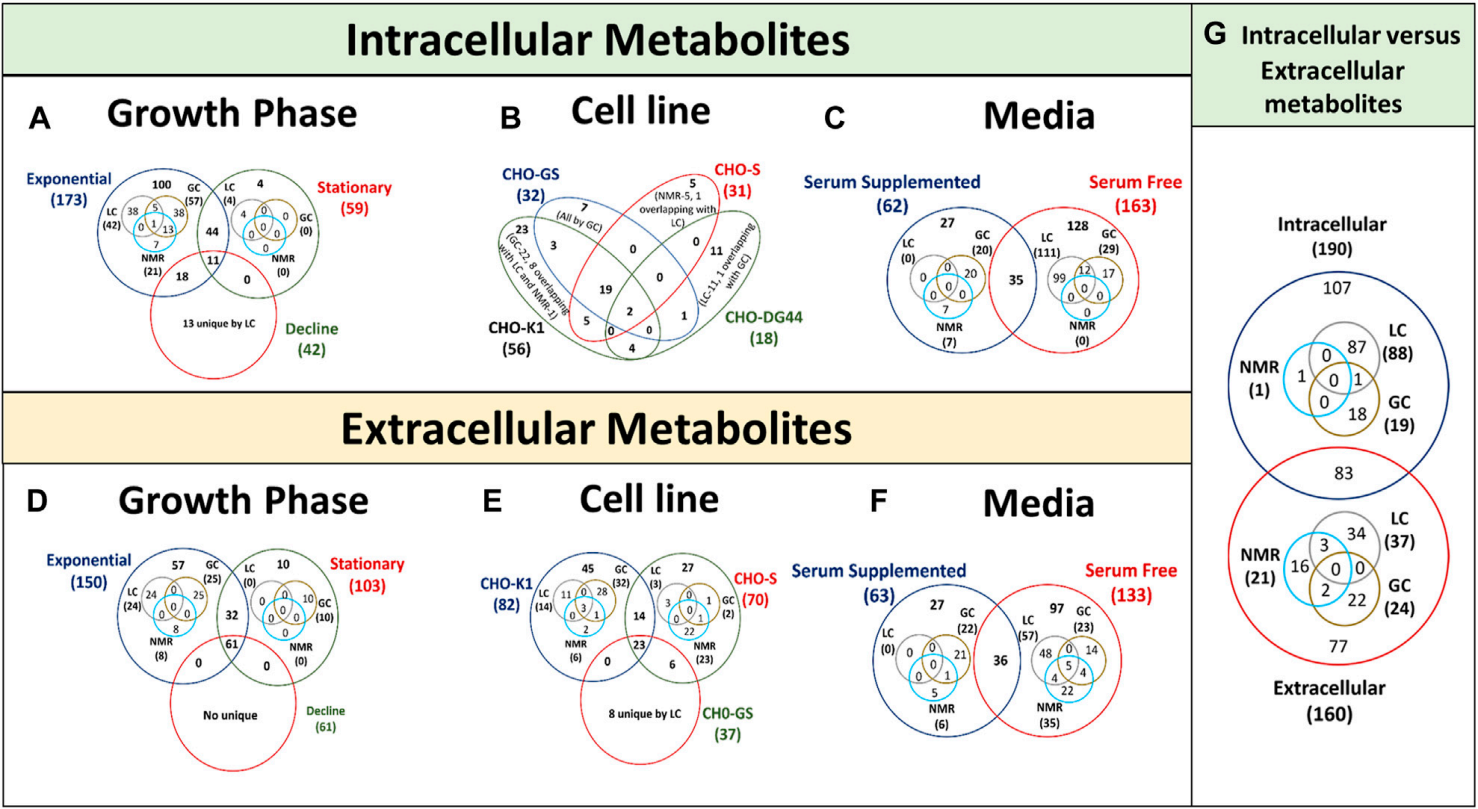
Venn diagrams showing overlap of intracellular and extracellular metabolites in CHO cultures identified using LC-MS, GC-MS and NMR. **(A, D)** represents data of intracellular and extracellular metabolites for different growth phases, **(B, E)** represents data of intracellular and extracellular metabolites for cell lines, **(C, F)** represents data of intracellular and extracellular metabolites for culture media and **(G)** represents data of intracellular and extracellular metabolites for Venn diagram is presented with the overlapping regions corresponding to the number of expressed metabolites present in more than one parameter type. The central region corresponds to the expressed metabolites present in all conditions of the same parameter.

Several studies have used metabolomics to compare the metabolic characteristics of high-growth and/or high-productivity cell lines to identify metabolites that accumulate in the cell or culture and inhibit growth (Figures 4A, D) and/or productivity. However, these studies vary in terms of CHO cell hosts (and clone) (Figures 4B, E), medium (and feed) (Figures 4C, F), type of recombinant protein being expressed and culture conditions limiting its application in other systems. As a result, it is unclear whether the metabolites identified in these studies are only relevant to a particular culture media, product and/or cell line, as each cell line may have different host cell proteins and metabolome under different media. Whereas, the need of the hour is the identification of more general/universal growth and/or productivity indicators that can be applied to multiple hosts and culture media for improved clone selection and/or process designing.

## 5 Evolving methods and future perspectives in CHO metabolomics

There have been a few significant technical advancements in the domain of metabolomics and in the understanding of the metabolomics pipeline that may facilitate better designing of future studies to investigate CHO cell behaviour in culture and get requisite knowledge to better regulate mammalian bioprocess for improved protein productivity.

### 5.1 Technological advancements

High-resolution mass spectrometry (HRMS) is an evolving method in CHO metabolomics that offers high accuracy and sensitivity in the detection of isobaric metabolite detection (Castelli et al., 2022). Isobaric metabolites represent the same molecular weight but different structures. This is a significant advantage over traditional MS methods, which cannot differentiate between isobaric compounds. Using HRMS, the glucose and galactose were reported to have a significant impact on CHO cell metabolism, while other sugars (such as GDP-Fucose, UDP-Glc/UDP-Gal, UDP-GlcNAc/UDP-GalNAc) had no or minor effect (Durrant et al., 2020). HRMS can also be utilized to analyse glycosaminoglycans (GAGs), which are complex carbohydrates that play a critical role in various biological processes (Torres CL. et al., 2023). Isotope labelling is another evolving method in CHO metabolomics which involves the incorporation of stable isotopes such as ^13^C and ^15^N into carbohydrates, for tracking the *in vivo* fate of carbohydrates in various metabolic diseases such as diabetes and obesity (Marin et al., 2004). This method shall be applied to study carbohydrate/amino acid’s metabolism in CHO-based bioprocessing also. Advancements in NMR techniques like 2D NMR also presents a chance to uncover signals that are overlapping in 1D NMR spectroscopy due to similar resonant frequencies and hence discover metabolites that were otherwise not resolved by 1D NMR (Mahrous and Farag, 2015). Besides, an emerging technique, Mass Spectrometry Imaging (MSI), is also a highly futuristic tool which enables untargeted investigations of a variety of samples sectioned into different spatial distributions (Liao et al., 2023). It has a capability to image thousands of molecules, such as metabolites, lipids, peptides, proteins, and glycans, in a single experiment without labelling. The information gained from MS and MSI can also be combinedly used for analysis and characterisation of biological samples.

### 5.2 Multiple analytical and/or sample preparation approach

An approach of combining multiple analytical platforms (NMR and MS) can be advantageous in order to improve the metabolome coverage. Integration of GC–MS, LC-MS and NMR has been shown to increase the metabolomics coverage of cells in culture (Bhinderwala et al., 2018; Fei et al., 2019; Zeki et al., 2020). Combining NMR and MS for metabolomics by using small chemical compound-treatments of *Chlamydomonas reinhardtii*, identified a total of 122 metabolites were detected (82 by GC-MS, 20 by NMR, and 20 by both); more than any single technique identified (Bhinderwala et al., 2018). Metabolomics of human urine samples using analytical platforms (NMR, GC-MS, LC-MS, ICP-MS and HPLC) identified a total of 445 metabolites consisting 209 by NMR, 179 by GC-MS, 12 by DFI/LC-MS/MS, 40 by ICP-MS and 10 by HPLC (Bouatra et al., 2013). Combining NMR with MS was reported to identify 21 altered metabolites between cancer and healthy controls, of which 13 were first time reported (Zhong et al., 2022). Combined NMR and MS also have advantages for isotope tracing and metabolic flux analysis (Shi et al., 2020). MS generally quantifies isotopic labelling distributions; even with MS/MS, it often does not give the specific labelling position, which can be detected using NMR. Besides, utilizing different methods of sample preparation/metabolite extraction has also been reported to improve metabolomic coverage. For example, in LC-MS of *Klebsiella pneumoniae*, 151 metabolites were identified in sample with metabolite extracted using Freeze thaw cycle (FTC), 103 by sonication cycle (SC) method and 132 metabolites by both (FTC + SC), enabling cumulative identification of 199 unique metabolites; more than any of the single method identified (Kumar et al., 2022). Similarly, in LC-MS of *Staphylococcus aureus*, a total of 116, 119, and 99 metabolites were identified in samples with metabolite extracted using the FTC, SC, and FTC + SC methods, respectively, leading to the identification of 163 metabolites cumulatively (Singh et al., 2023). This indicates that each method of metabolite extraction also has its own biasness (Sowa et al., 2020; Singh et al., 2023). Therefore, utilizing multiple methods of metabolite extraction with multiple analytical methods (LC-MS/GC-MS and NMR) will present a clearer and more accurate picture of the metabolic profile due to increased metabolomic coverage and help to overcome the limitations of individual techniques/methods.

### 5.3 Improved bioinformatics solutions

There is an urgent need for improved and user-friendly bioinformatics tools to analyse metabolomic data and integrate this knowledge with other ‘omic’ approaches. As of now, data driven mathematical modelling has proven beneficial for optimizing media composition, culture parameters, metabolites, growth and productivity from the CHO cell lines (Galleguillos et al., 2017). Commonly used modelling approaches performs non-steady-state kinetics based metabolic flux analysis (MFA) and flux balance analysis (FBA) using system biology and machine learning (Nolan and Lee, 2012). Recently, a model constructed using production rates of metabolites as a function of specific growth rates from a 2L small-scale culture as training set were observed to identify metabolic phases and predict cell metabolism and productivity of 2000L production scale (Ben Yahia et al., 2017). However, data-driven models are challenging due to data scarcity and heterogeneity and may be of limited use to extrapolate beyond the training set (Danzi et al., 2023; Gong et al., 2023). Hence, hybrid models combining fundamental kinetics with data-driven approach were developed which included impurities generated by the cell (host cell proteins and DNA content released due to cell death) during the process along with description of cell viability, mAb production, glucose, and lactate concentrations (Okamura et al., 2022). The model was observed to yield higher accuracy than both the kinetic or statistical modules alone. Genome-scale metabolic models (GeMs), which integrate the knowledge from genomics, transcriptomics, epigenomics, proteomics and metabolomics, provide detailed information about biochemical reactions networks that compose cellular metabolism and offers potential to connect molecular networks to the observed phenotype (Rejc et al., 2017). In the line, a number of metabolic pathways were reconstructed and associated with >1,700 genes in CHO genome as a resource for GeM to predict the growth and productivity characteristics of the CHO cells (Hefzi et al., 2016). A GeM analysis of a wild-type and antibody producer CHO cell line revealed extensive transcriptional re-wiring of DNA damage repair and cellular metabolism with the genomic data, supported by substantial increase in energy metabolism in the producer cells by the transcriptomic data and elevated long chain lipid species (potentially facilitating the protein transport and secretion requirements) by metabolomic data establishing that observation from different omics data sets to be significantly overlapping and complementary (Yusufi et al., 2017). GeMs constructed using unconventional uptake-rate objective functions (primarily considering the availability of CHO-specific essential nutrients in the media) instead of conventional biomass objective function have been shown to be more accurate and able to metabolically distinguish different CHO cells compared to predictions based on conventional biomass objective functions (Chen et al., 2019). Recently, CHOmpact, a reduced metabolic model, was reported to deploy robust and nonlinear optimization, compute physiologically consistent flux distributions and enhanced interpretability of simulation (Jiménez del Val et al., 2023). However, implementation of GeMs based approaches in CHO based bioprocess is still limited. In overall, as of now, no single method or approach is comprehensive, but rather, complementary.

### 5.4 Comprehensive metabolomics

More targeted efforts are required to investigate the intracellular metabolites of the most effective cell phenotype in the bioprocess as extracellular metabolic profiles alone typically fail to explain cellular behaviour (high-growth or -productivity) in the production culture (Dietmair et al., 2012). A total of 107 unique intracellular metabolites, 77 unique extracellular metabolites and 83 common metabolites have been reported in different CHO based metabolomics studies performed using LC-MS, GC-MS and NMR (Figure 4G). The intracellular metabolite enrichment could be due to their uptake from culture media as it is (nutrient) or as its precursor. Therefore, analysing only extracellular or intracellular metabolome may remain unable in elucidating the detailed metabolomic process suggesting their complementarity. Integrating quantitative extracellular metabolomic profiles with intracellular metabolic profiles and flux states might enable to better understand the metabolomic variations and candidate flux distributions in CHO bioprocess. Hence, intracellular and extracellular metabolic profiling shall be performed together to achieve a holistic picture of metabolomic pathways working together.

## 6 Conclusion

CHO cells are the most preferred cell lines for industrial production of protein biotherapeutics. Significant improvement in the yield of such products has been witnessed over time, primarily because of improved media and bioprocess design. However, these products still remain costly and hence demand further improvement in the performance of CHO cells in the bioprocess. This could only be achieved by improving our understanding of the different cellular phenotypes, good/bad-grower and/or producer CHOs, in the culture.

Metabolomics is a promising approach in the bioproduction field, as it detects the downstream products (metabolites) of the other ‘omics’ sciences - genomics, transcriptomics and proteomics and is believed to mirror the cellular phenotype more accurately. Despite the limited utilization in the domain, metabolomics has contributed significantly in designing the media and feed to achieve improved cell growth and overall productivity. Initially, increased consumption of glucose and glutamine were known to increase generation and accumulation of lactate and ammonia in culture that are well-known to inhibit the cell-growth and productivity in culture. However, overtime, with increasing efforts of investigating CHO metabolome, our knowledge of cell metabolism in bioprocess has improved and as a result, several metabolites and pathways that are associated with regulation of cell growth and productivity in culture, have been identified. For example, increased glycolytic pathway is shown to support the cell growth in exponential phase, whereas increased TCA cycle, oxidative phosphorylation and glutathione pathways have been shown to slow-down cell growth and -productivity in CHO bioprocess. The availability of amino acids, specifically aspartate, citrate and histidine, has been shown to be associated with cell-specific productivity in culture. Recent technical advancement and our experience with the technology suggest that metabolomics can help in identifying not only extracellular growth- and/or productivity-regulating metabolites to further improve culture media and feed but also intracellular metabolites and pathways that actually performs to achieve improved growth and productivity from production culture, providing the targets for rational cell engineering. However, multiple analytical and sample preparation methods must be employed to achieve higher metabolomic coverage and minimize the chances of missing out the identification of key-regulator metabolites during metabolomics-based investigations. Taken together, metabolomics-based investigations offer great potential in improving CHO-based bioprocesses’ performance and hence demand more profound investigation.
